# Aging With HIV: Inflammation Is Associated With Pain, Poorer Physical Function, and Frailty

**DOI:** 10.1093/geroni/igaa057.1072

**Published:** 2020-12-16

**Authors:** Heather Derry, Carrie Johnston, Chelsie Burchett, Eugenia Siegler, Marshall Glesby

**Affiliations:** Weill Cornell Medicine, New York, New York, United States

## Abstract

With advances in antiretroviral therapies, people living with HIV have life expectancies similar to their HIV-negative peers. Yet, they experience elevated multi-morbidity that can compromise quality of life as they age. Links between inflammation and accelerated aging may inform interventions, but these links are understudied in older adults with HIV. We investigated cross-sectional relationships between inflammation, well-being, and geriatric syndromes among 161 HIV-positive older adults. Participants provided fasting blood samples (for serum cytokines and CRP) and completed surveys (MOS-HIV; falls) and cognitive (MoCA) and frailty assessments (using Fried criteria). Adjusted linear and logistic regression models tested relationships between inflammatory markers and age-related health outcomes, controlling for age, gender, BMI, race, comorbidity burden, statin use, and smoking status. 93% had suppressed viral load. 11% had CRP levels suggesting possible acute illness (>10 mg/L) and were excluded from analyses. Participants with higher IFN-γ reported greater pain (p=0.003), greater cognitive complaints (p=.02), and worse physical function (p=0.04), than those with lower IFN-γ. Similarly, higher IL-6 levels were related to worse physical function (p=0.01) and slightly greater cognitive complaints (p=0.06), but were not significantly related to pain in adjusted models. Compared to those with lower IL-6, those with higher IL-6 levels were more likely to be frail (p=0.04). CRP was not significantly related to these outcomes. Six-month fall history and objective cognitive scores were not significantly related to the assessed inflammatory markers. Our results illustrate key, expected links between inflammatory processes, frailty, physical function, and pain among older adults with HIV.

